# Congenital giant right atrial aneurysm: Surgical reduction plasty and ablation during infancy

**DOI:** 10.1016/j.xjtc.2026.102313

**Published:** 2026-03-18

**Authors:** Luigi Di Pasquale, Michael Reinehr, Christian Balmer, Matthias Gass, Hitendu Dave

**Affiliations:** a Congenital Cardiovascular Surgery, University Children's Hospital Zürich, Zürich, Switzerland; b Department of Pathology, University Hospital Zürich, Zürich, Switzerland; c Pediatric Cardiology, University Children's Hospital Zürich, Zürich, Switzerland

**Figure:**
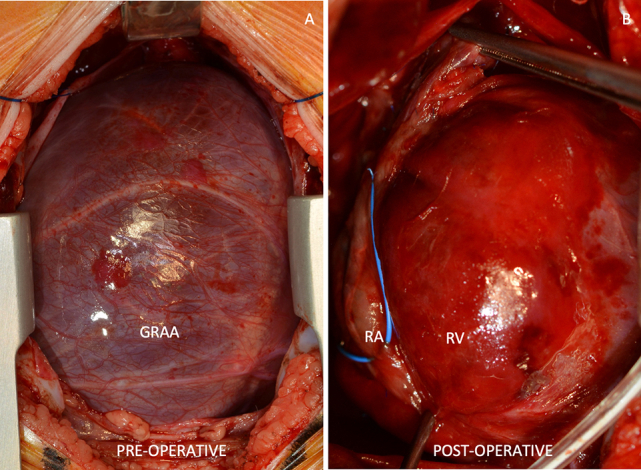
Giant RA aneurysm: before (A) and after (B) surgical reduction plasty.

**Central Message**

Surgical overlapping reduction plasty and limited right atrial (RA) maze of a giant RA aneurysm (GRAA) during infancy is innovative, safe, and effective.

A few sporadic cases of giant right atrial aneurysm (GRAA) have been reported.^1^ Herein, we report overlapping reduction plasty with limited right atrial (RA) maze in 2 infants (Table 1). The Cantonal Ethics Committee waives the need for formal approval for case reports. General consent for anonymous use of data was provided by the patients’ parents.

**Table 1:** Demographics

Perioperative variables	Child 1	Child 2
Fetal diagnosis	28 GW/F The CT ratio as well as ratio of RA to heart increased from 0.5 to 0.7 and from 0.57 to 0.7, respectively, during pregnancy.	21 GW/F
Weight at birth, kg	3.3	3
Diagnosis (echocardiography, MRA)	GRAA, ASD II, PDA, TR+	GRAA, ASD II, PDA, TR +++ (dysplastic tricuspid valve causing severe regurgitation); first-degree AV block
Size of the GRAA, mm (z score)	50 (+10.8) × 37 (+8.3)	50 (+11.3) × 35 (+8.2)
Symptoms	Intra-atrial re-entry tachycardia (DD flutter) requiring cardioversion and antiarrhythmic therapy (flecainide-propranolol on day 5)	Cardiopulmonary impairment with respiratory distress syndrome requiring invasive respiratory support and catecholamine at 2 min postnatal
Indication for operation	Arrythmias; thromboembolic risk (MRA showed low-velocity vortices and areas of stasis in RAA)	Extrinsic compression of LV (persistent CPAP) Severe TR; marginal cardiac output - ischemic colitis; thromboembolic risk
Genetic		Maternally transmitted variant of *LAMA4* gene of unclear significance c.2153B 2161del p.(Gly71B_GlnT2ldelinsGlu) in LAMA4-Gen
Age and weight at operation	49 d/5 kg	26 d/3.1 kg
Surgical procedure	GRAA-reduction plasty and ablation, ASD closure, PDA ligation	GRAA-reduction plasty and ablation, fenestrated ASD closure, PDA ligation Tricuspid reconstruction
Intraoperative findings		Right coronary artery course across the aneurysm
CPB/aortic crossclamp time, min	138/55	97/63
Ventilation, d	1	7
ICU stay, d	2	8
Hospital stay, d	10	64 (GI issues)
Rhythm at discharge	Sinus rhythm	Sinus rhythm
Reduction in CT ratio (preoperative to postoperative) (Figure E1)	0.70 to 0.45	0.77 to 0.52

*GW*, Gestational weeks; *F*, female; *CT*, computed tomography; *RA*, right atrial/atrium; *MRA*, magnetic resonance angiography; *GRAA*, giant right atrial aneurysm; *ASD*, atrial septal defect; *PDA*, patent ductus arteriosus; *TR*, tricuspid regurgitation; *+/++/+++*, grade 1, 2, 3 regurgitation; *AV*, atrioventricular; *DD*, differential diagnosis; *RAA*, right atrial aneurysm; *LV*, left ventricle; *CPAP*, continuous positive airway pressure; *CPB*, cardiopulmonary bypass; *ICU*, intensive care unit; *GI*, gastrointestinal; *CT* ratio, cardiothoracic ratio.

Once on cardiopulmonary bypass (CPB), the GRAA collapsed and the patent foramen ovale could be seen through it (Figures 1 and 2). The aorta was crossclamped and cardioplegia was administered. The central strip of the aneurysmatic wall was excised; medial and lateral flaps were then fashioned (Figures 1 and 2). A partial RA maze was performed using surgical and radiofrequency ablation. A superior vena cava to inferior vena cava ablation line was created along the crista terminalis using bipolar electrocautery. The thin wall, which tore apart at places, was surgically sutured. Cavotricuspid isthmus ablation was performed using a transpolar pen (Isolator Transpolar Pen MAX1; AtriCure) (Figures 1 and 2). The LV was deaired, the patent foramen ovale closed, and aorta was unclamped. The medial flap was sutured to the crista terminalis. The lateral flap was loosely fixed to buttress the thin wall, so as to avoid hematoma formation between layers (Figures 1 and 2).

**Figure 1:**
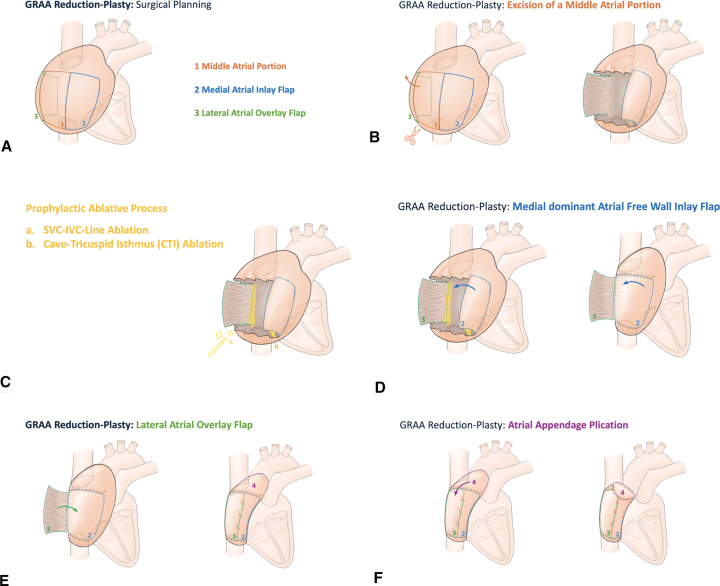
A-F, Surgical technique. *GRAA*, Giant right atrial aneurysm; *SVC*, superior vena cava; *IVC*, inferior vena cava.

**Figure 2:**
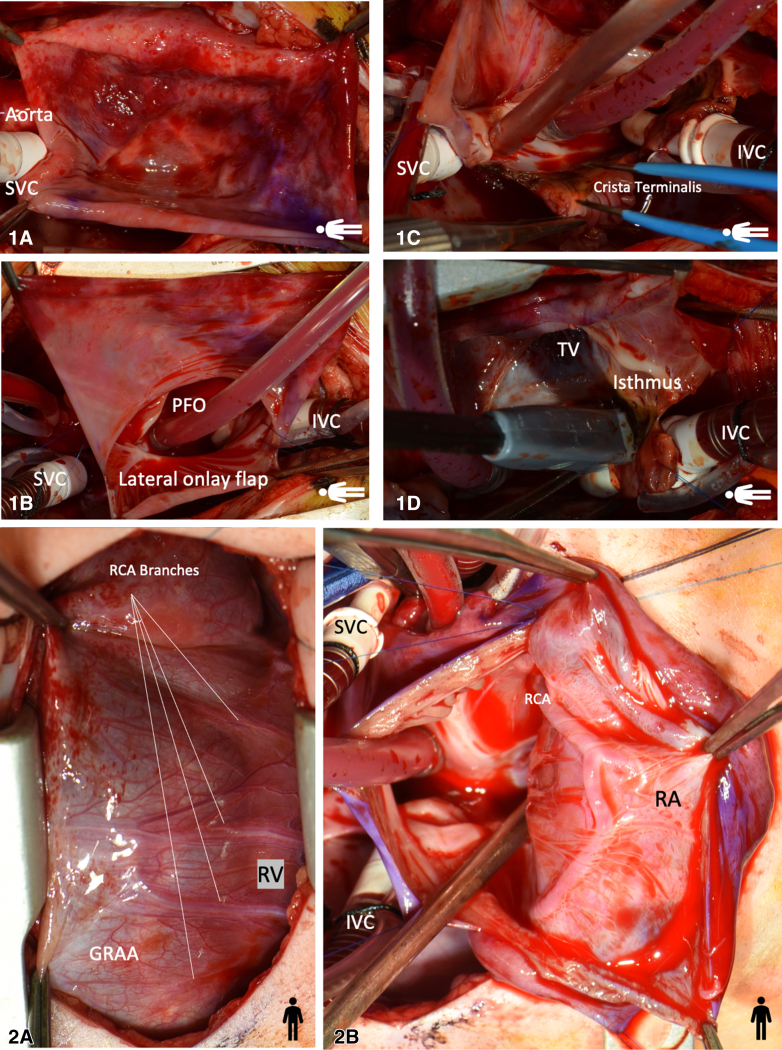
Intraoperative images: Child 1 (1A): Collapsed GAAA on CPB, (1B): creation of a right atriotomy flap, (1C): SVC-IVC ablation line, and (1D): isthmus ablation. Child 2 (2A): RCA twigs crisscrossing GRAA, (2B): coronary traversing the aneurysm. *GRAA*, Giant right atrial aneurysm; *CPB*, cardiopulmonary bypass; *SVC*, superior vena cava; *IVC*, inferior vena cava; *RCA*, right coronary artery; *PFO*, patent foramen ovale; *TV*, tricuspid valve.

The aneurysmatic (dysfunctional) right atrial aneurysm was obliterated by suturing at the base and folding over itself. A resection (with proximity to the right coronary artery [RCA]) was considered dangerous. A close watch on the RCA was kept during resection and suturing, to avoid an unexpected, potentially dangerous, event. Tricuspid reconstruction was performed when required by commissural plasty. Weaning from CPB was uneventful in both patients. Sinus rhythm ensued without the need for pacing. Postoperative echocardiography demonstrated a harmonious reduction of the GAAA. Video 1 depicts each step in detail. Follow-up is shown in Table 2.

**Table 2:** Follow-up

Follow-up	Child 1	Child 2
Follow-up duration, mo	53	18
Weight at follow-up, kg	20.4	8.9
Symptoms at follow-up	None	None
Rhythm at follow-up	Sinus rhythm alternating with atrial rhythm without medications Normal chronotropy. No arrhythmias observed.	Sinus rhythm (without medication) Diuretics for TR
Echocardiographic findings at follow-up	Normal biventricular function. Contractile RA.	Normal Biventricular function. Contractile RA. TR+/++ (due to apical displacement of anterior leaflet)
RA diameter, mm (z value)	42 (+3.17) × 30 (+0.57)	42 (+5.91) × 23 (+0.72) RCA crossing the aneurysm

*TR*, Tricuspid regurgitation; *RA*, right atrial/atrium; *RCA*, right coronary artery.

## Discussion

**Figure 3:**
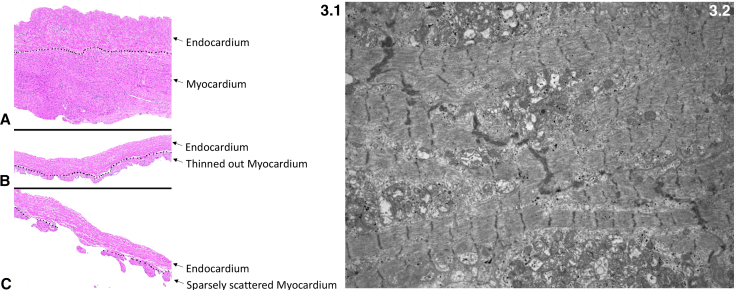
Histopathology. (1) Hematoxylin and eosin stain (×50). A, Normal RA wall. B, Thinned-out RA wall (*black dotted line* separates endothelium from myocardium). C, GRAA—extremely thinned out areas with missing myocardium—direct apposition endocardium to epicardium. (2) Electronic microscopy (GRAA) showing extreme rarefication of myocardium. Myofibrillar junction demonstrated intact desmosome and fascia adherents. Intracellular organelles appeared normal. *RA*, Right atrium; *GRAA*, giant right atrial aneurysm.

GRAA is a rare anomaly with an unknown etiology. Case 1, being isolated, is clearly a primary GRAA, but case 2, because of its association with (dysplastic) tricuspid regurgitation, is not straightforward. In concordance with Wang and colleagues,^2^ this presentation reflects the coexistence of 2 distinct but interrelated cardiac pathologies—GRAA and tricuspid dysplasia. It also differs from Ebstein anomaly, because the tricuspid valve had 3 leaflets with normal septal insertion, offset relative to mitral valve, and no ventricular atrialization. This was supported by the macro- and microscopic anatomic findings, including a markedly enlarged right atrium with a paper-thin atrial wall and regions containing only a minimal myocardial layer, features characteristic of primary atrial aneurysmal disease (Figure 3). In contrast, secondary forms, observed in other patients (not included in this report), involved giant RA (severe tricuspid regurgitation) with substantially thicker RA wall. Moreover, case 2 had an anomalous RCA across the aneurysm, which has been reported in cases of GRAA.^3^

The presentation, progression, and extent of this anomaly varies, and hence the timing and technique of repair have remained individualized. The clinical presentation ranges from asymptomatic patients to those diagnosed as the result of signs of compression, arrhythmias, and sudden death. Although symptoms drive the indication for intervention,^4^ spontaneous regression of GRAA is not known. Conservative management of small RA aneurysms is justified; however, GRAA (Z +10 to +11) may justify an aggressive approach. Although the majority of GRAAs are diagnosed during infancy, a significant number also present in adulthood.^4^ This may change with the greater availability of echocardiography. Although published mortality of reported cases is approximately 5%,^5^ this number may not capture the patients who die before presentation. Considering the simplicity and low risk of the surgical correction, prophylactic operation in asymptomatic massive GRAA may be considered. This would help reduce the risk of life-threatening arrhythmias, thromboembolic events, and right heart failure. Anticoagulant therapy should be given to asymptomatic patients.

The safety of modern CPB today appears to override the temptation of avoiding CPB in GRAA with an extremely thinned-out wall.^1^^,^^4^ The use of cardioplegia depends on the presence of intracardiac shunts and the necessity of concomitant valve repair. Moreover, the risk of RCA distortion cannot be overemphasized. Extensive atrial suture lines on a thin atrial wall are a substrate for arrhythmias necessitating medical-ablative therapy.^4^ Thus, our concept of overlapping plasty with prophylactic RA maze is appealing.^5^ Although more experience is necessary, our limited experience of thriving children without antiarrhythmics and an atrial kick at follow-up portends well for the long-term outcome.

## Conflict of Interest Statement

The authors reported no conflicts of interest.

The *Journal* policy requires editors and reviewers to disclose conflicts of interest and to decline handling or reviewing manuscripts for which they may have a conflict of interest. The editors and reviewers of this article have no conflicts of interest.

## Supplementary Data

Video 1 Step by step depiction of surgical reduction plasty and ablation. Video available at: https://www.jtcvs.org/article/S2666-2507(26)00120-3/fulltext.
