# Supplementary figures and images for: Congenital giant right atrial aneurysm: Surgical reduction plasty and ablation during infancy
Source: JTCVS Tech. 2026 Mar 18;37:102313. doi: 10.1016/j.xjtc.2026.102313 (PMC13261244; doi:10.1016/j.xjtc.2026.102313)

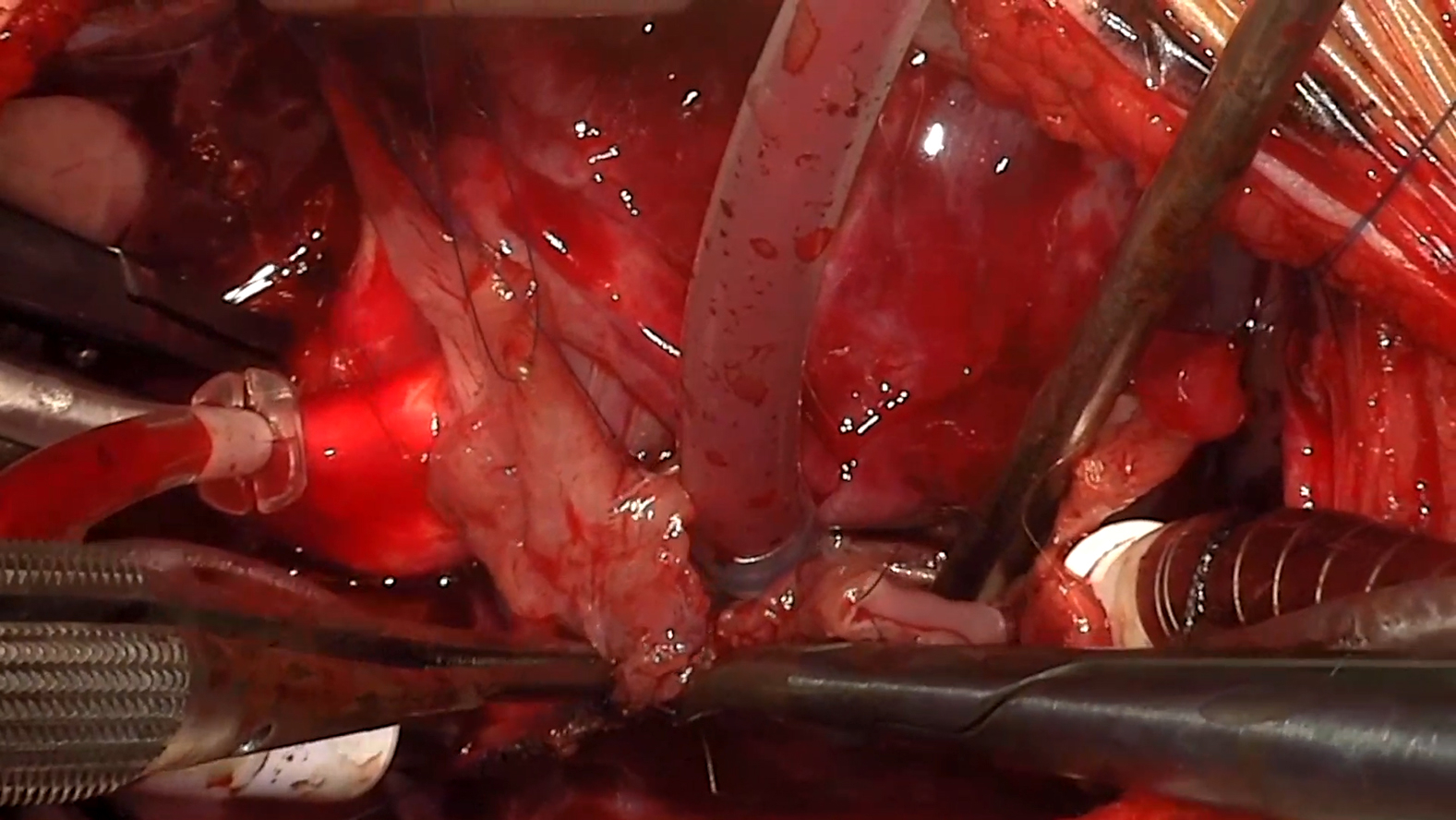

Supplement: Video 1 — Step by step depiction of surgical reduction plasty and ablation. Video available at: https://www.jtcvs.org/article/S2666-2507(26)00120-3/fulltext. [file fx2.jpg]
