# Supplementary material for: Congenital giant right atrial aneurysm: Surgical reduction plasty and ablation during infancy
Source: JTCVS Tech. 2026 Mar 18;37:102313. doi: 10.1016/j.xjtc.2026.102313 (PMC13261244; doi:10.1016/j.xjtc.2026.102313)
Supplement: Online Data Supplement [file mmc2.pdf]

Supplementary Figures

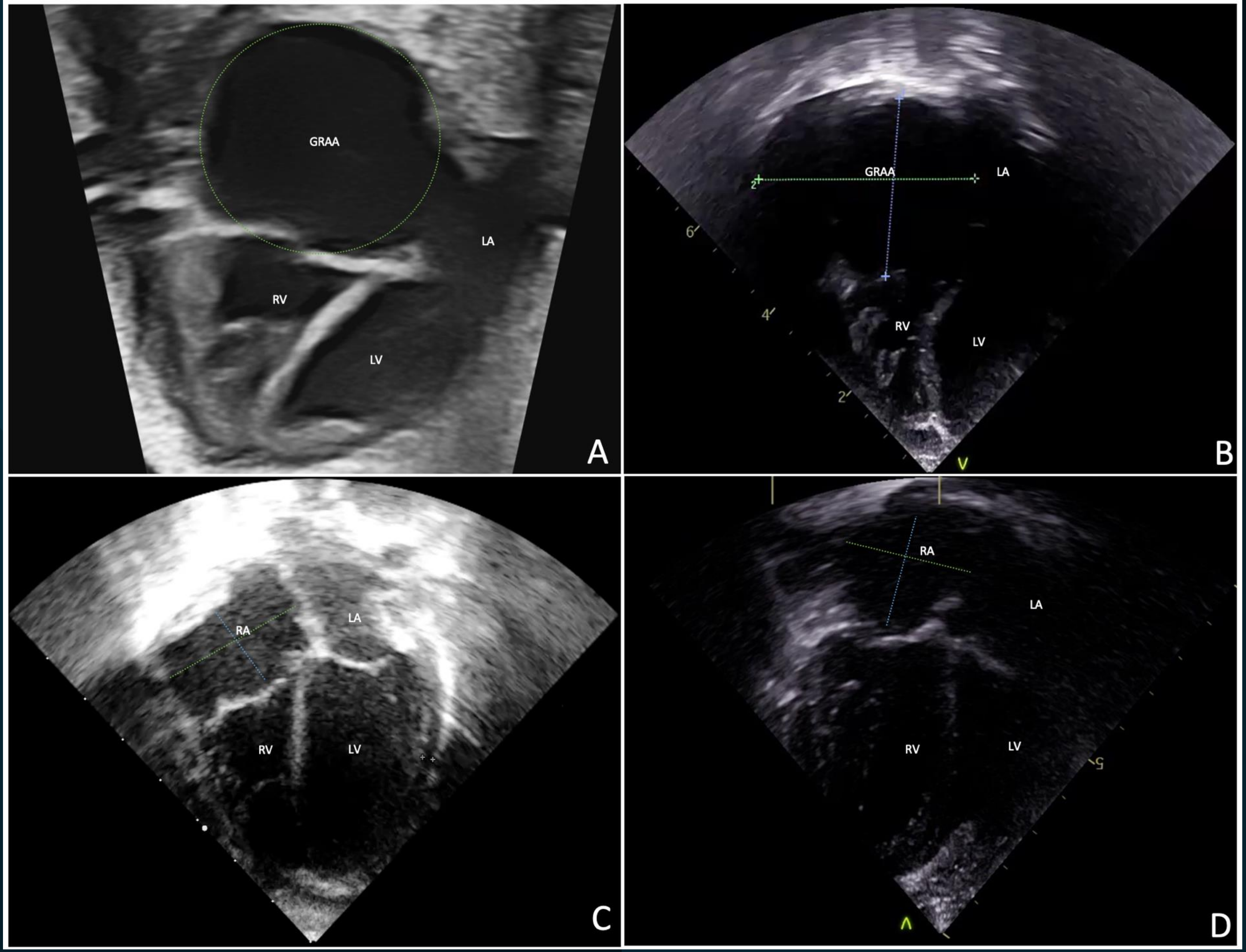

Supplementary Figure 1. Chronological Transthoracic Echo of GRAA (Child 1)
A. Prenatal (28 3/7 Gestational Age); B. Preoperative; C. Postoperative; D. Follow-Up

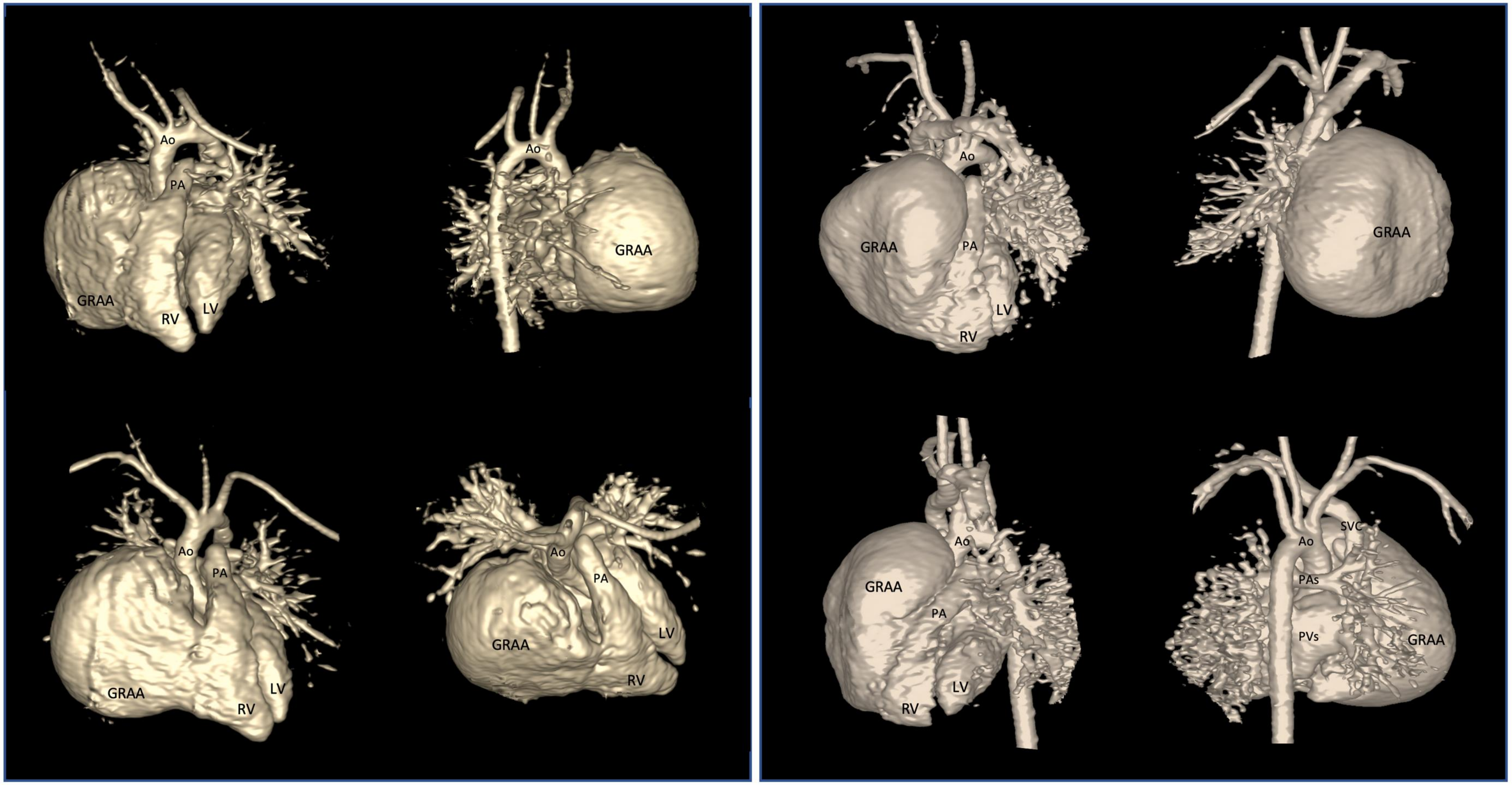

Supplementary Fig. 2

Magnetic Resonance Imaging (MRI) – 3D Reconstruction: Child 1, Child 2

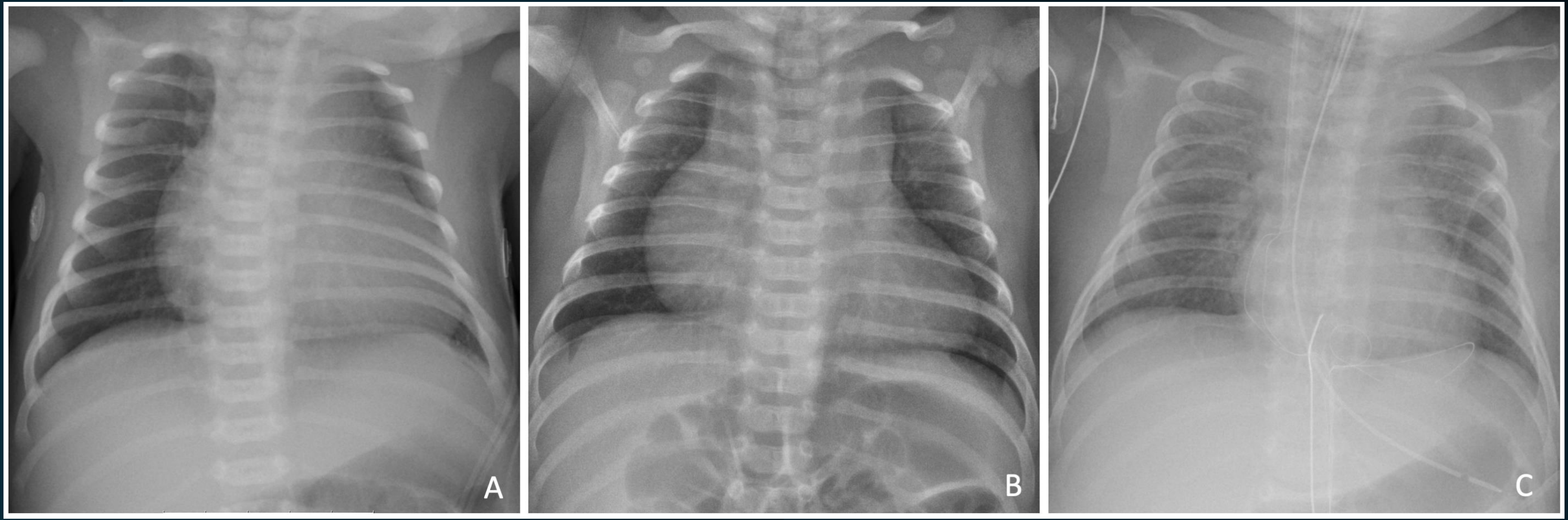

Supplementary Fig. 3

Chest radiography (Anterior Posterior View): A. At Birth; B. Preoperative; C. Postoperative

The chest X-ray showed a cardiothoracic ratio reduction from 0.7 to 0.45.

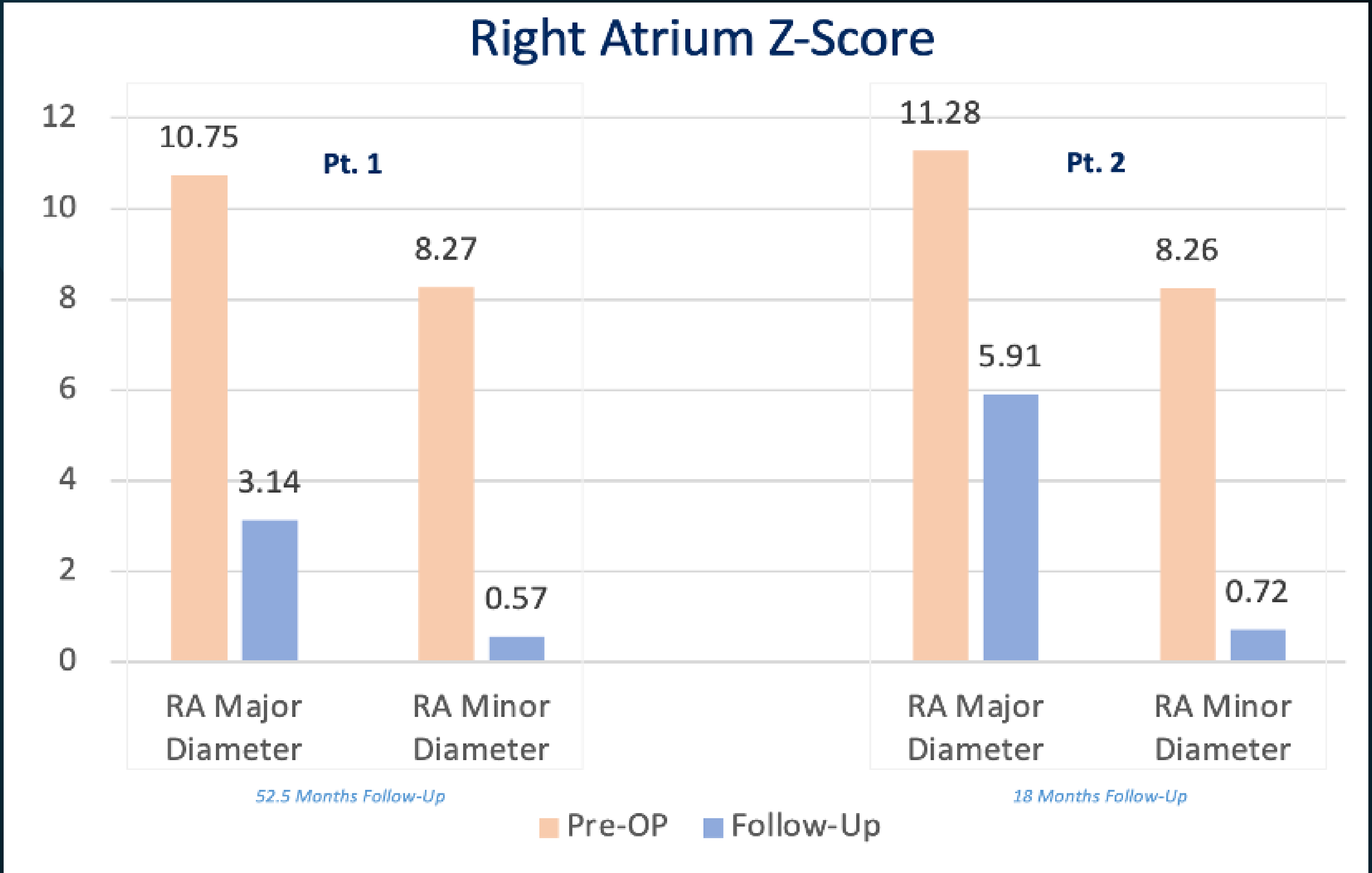

Supplementary Fig. 4

Right atrium Z-score: Pre-operative and at follow-up
